# Flower induction, microscope-aided cross-pollination, and seed production in the duckweed *Lemna gibba* with discovery of a male-sterile clone

**DOI:** 10.1038/s41598-017-03240-8

**Published:** 2017-06-08

**Authors:** Lili Fu, Meng Huang, Bingying Han, Xuepiao Sun, K. Sowjanya Sree, Klaus-J. Appenroth, Jiaming Zhang

**Affiliations:** 1 Institute of Tropical Bioscience and Biotechnology, MOA Key Laboratory of Tropical Crops Biology and Genetic Resources; Hainan Bioenergy Center, CATAS, Haikou, Hainan Province 571101 China; 2 Department of Environmental Science, Central University of Kerala, RSTC, Padanakkad, 671314 Kerala India; 3 Institute of Plant Physiology, University of Jena, Dornburger Str. 159, 07743 Jena, Germany

## Abstract

Duckweed species have a great potential to develop into fast-growing crops for water remediation and bioenergy production. Seed production and utilization of hybrid vigour are essential steps in this process. However, even in the extensively-studied duckweed species, *Lemna gibba*, flower primordia were often aborted prior to maturation. Salicylic acid (SA) and agar solidification of the medium promoted flower maturation and resulted in high flowering rates in *L. gibba* 7741 and 5504. Artificial cross-pollination between individuals of *L. gibba* 7741 yielded seeds at high frequencies unlike that in *L. gibba* 5504. In contrast to clone 7741, the anthers of 5504 did not dehisce upon maturation, its artificially released pollen grains had pineapple-like exine with tilted spines. These pollens were not stained by 2,5-diphenylmonotetrazoliumbromide (MTT) and failed to germinate. Therefore, clone 5504 is male sterile and has potential application with respect to hybrid vigour. Moreover, pollination of flowers of 5504 with 7741 pollen grains resulted in intraspecific hybrid seeds, which was confirmed by inter-simple sequence repeat (ISSR) markers. These hybrid seeds germinated at a high frequency, forming new clones.

## Introduction

Lemnaceae, commonly known as duckweeds, is a family of small aquatic monocotyledonous plants that comprise five genera and 37 species^1, 2^. They represent the smallest flowering plants with reduced morphology^3^. Duckweeds can rapidly assimilate nutrients such as nitrogen and phosphate from municipal, industrial, and agricultural wastewaters and have shown great value in wastewater remediation^4–9^. The biomass of duckweeds may contain high starch content and could be used to produce bioethanol and biobutanol^10–14^. Some duckweed clones contain high protein content, and have been used as high quality livestock feed^15–17^. Even the duckweed biomass accumulated on wastewaters may be used for feed production^18–20^.

*Lemna gibba* is one of the duckweed species that has been widely used in research and applications. *L. gibba* is very efficient in removal of organic and inorganic nutrients from domestic and industrial wastewater^21–24^, as well as heavy metals such as lead and uranium^25^. *L. gibba* was also found to be efficient in removal of harmful organisms such as *Giardia cysts*, *Cryptosporidium oocysts*, faecal coliforms and coliphage from wastewater^26^. It can also be used in bioassays of toxicity of industrial waste^21^.

**Figure 1:**
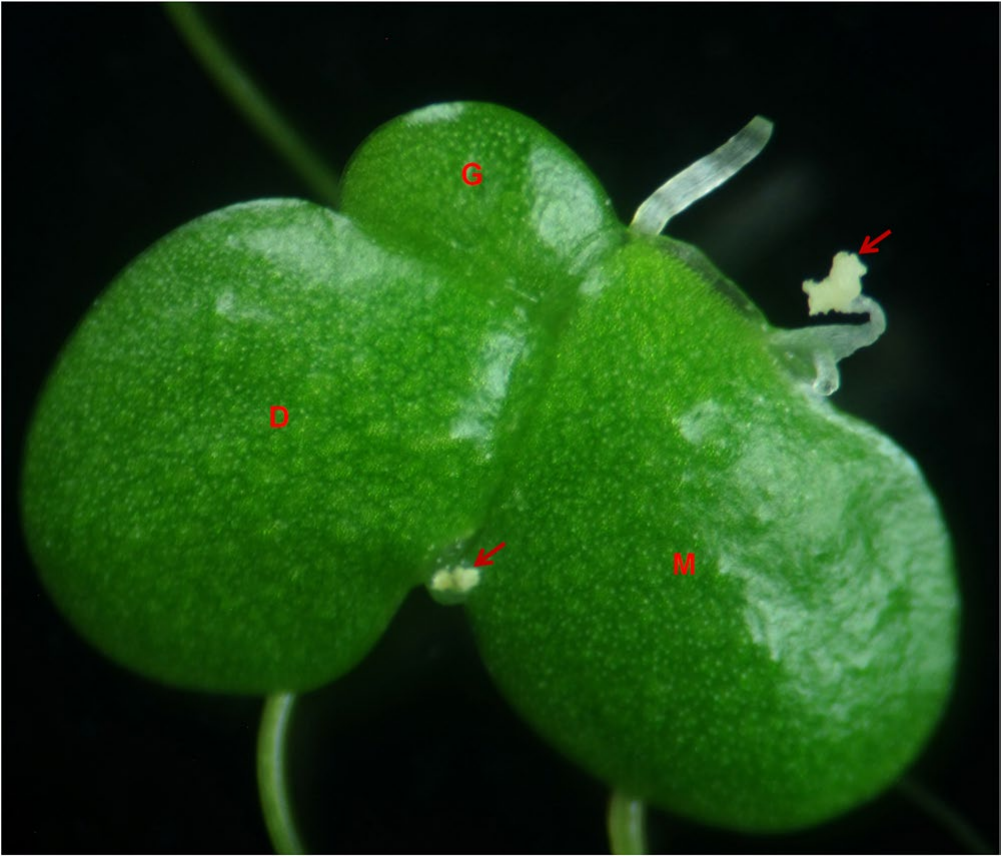
*L. gibba* plant with natural flowers. Red arrows indicate flowers; M, mother frond; D, daughter frond; G, granddaughter frond.

The potential of duckweeds in commercial applications has brought forward a need for domestication and the breeding of elite varieties serving different purposes. Seed production and application of hybrid vigour have an enormous importance in making duckweed a future crop plant. However, duckweeds reproduce mainly vegetatively. In this process, mother fronds form genetically identical daughter fronds that are therefore called a clone. Plants collected from different places are in most cases genetically different from one another and form different clones. Flowering is a rare event in nature and in the laboratory cultures in most duckweed species^27, 28^. Natural flowers are morphologically identical to those induced artificially (Fig. 1). Artificial induction of flowering and seed production in duckweeds is therefore needed for future efforts to breed new duckweed varieties by cross-pollination to improve their efficiency.

Many efforts have been made to induce flowering in different duckweed species. Salicylic acid (SA) is one of the most important plant hormones used in duckweed flower induction. SA induced flower formation in *L. gibba* G3 and *L. paucicostata* LP6 (valid term *L. aequinoctialis*), and the continuous presence of SA was found to be essential to obtain maximum flowering^29^. SA induced flowering in *L. gibba* G3 in coordination with day length^30, 31^. Flower promotion was obtained with day lengths of more than 9 h, but never with less than 8 h. Salicylic acid treatment caused a shift in the critical day length of about 2 hours^31^. Benzoic acid is another chemical that is effective in flower induction. When *L. gibba* G3 was grown on 1/10 M medium or 1/2 H medium under long day conditions, addition of benzoic acid to either media led to a flowering response, which was inhibited by the plant hormones IAA, GA3, ABA, and zeatin^32, 33^.

Analysis of flower induction in previous reports was based on dissecting the budding pockets of *L. gibba*. Fronds with flower primordia, i.e. immature flowers, visible under a dissecting microscope were usually considered as flowering^31, 32, 34^, whereas no data on induction of mature flowers are available up to date to the best of our knowledge. However, only mature flowers can be used for cross-pollination for the purpose of breeding. In this study, we present efficient methods to induce the formation of mature flowers and production of intraspecific hybrid seeds in *L. gibba*. For that purpose, two geographically distinct *L. gibba*, clones 7741 (origin: Italy, better known by the older term G3) and 5504 (origin: China), and one clone of *L. minor*, clone 7210 (origin: Japan) were investigated. The genetic background of these clones was investigated by barcoding according to the described methods^35–37^ (Supplementary material, Fig. S1). From clone 7741, seeds were produced by artificial self-pollination. Moreover, intraspecific hybrid seed production by pollen transfer between different clones of *L. gibba* under a microscope was successfully carried out for the first time. This strategy will be used in breeding programmes in the future to make duckweed a crop plant. Moreover, the analysis of pollen viability and cross-pollination led to the identification of a male-sterile line of *L. gibba* which is the first report in Lemnaceae family.

## Results

### Flower maturation in *L. gibba* is limited in conventional culture media

**Figure 2:**
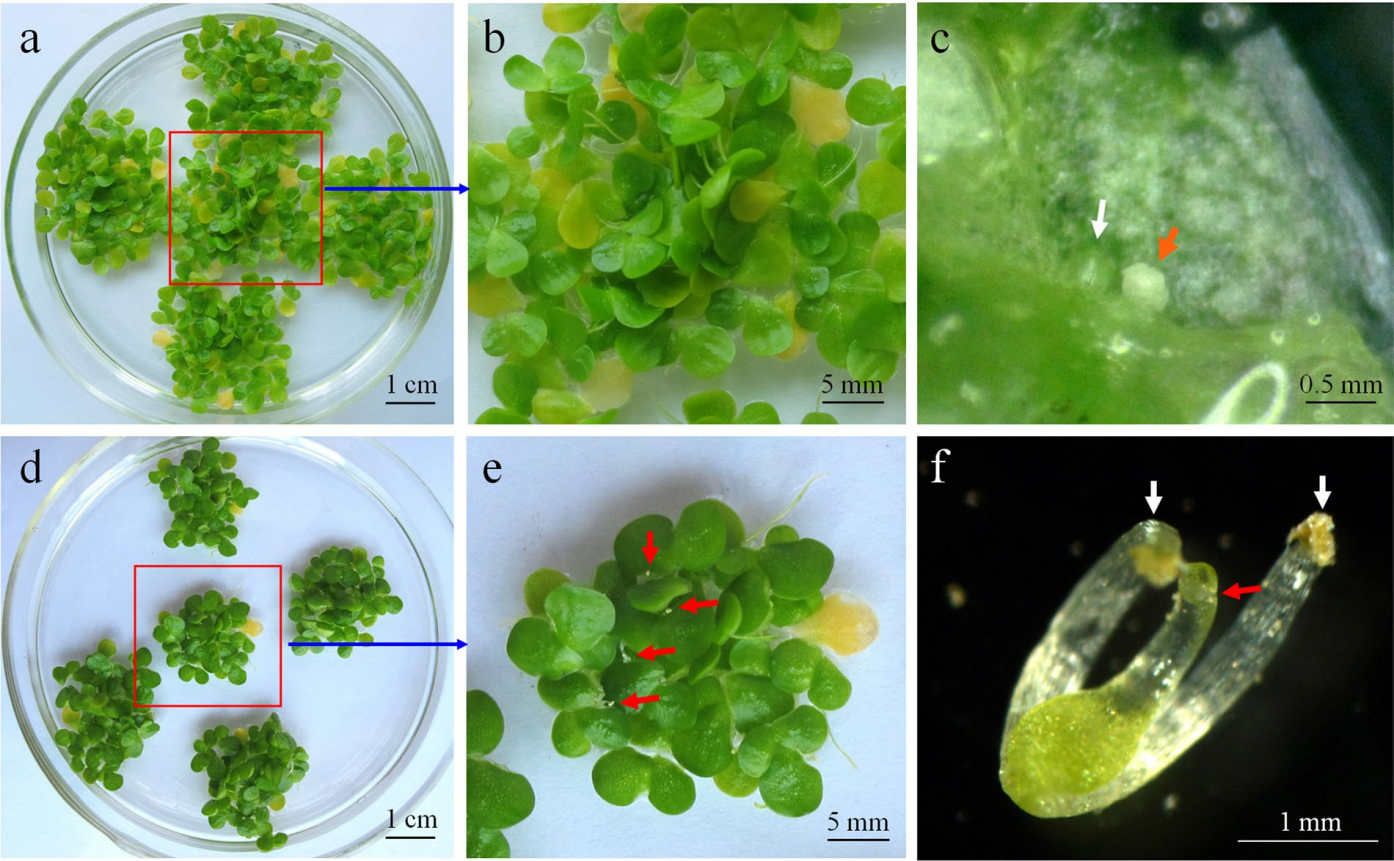
Comparison of *L. gibba* clone 7741 plants grown in media without ((**a**–**c**); upper row as a control) and with ((**d**–**f**); bottom row) salicylic acid (SA). (**a**) Control fronds grown in medium without SA showing high amount of biomass 15 days after inoculation. (**b**) Magnification of the *red-squared* region in (**a**) showing the absence of flowers. (**c**) Dissected budding pocket of a frond growing on SA-free medium; the white arrow indicates frond primordium, and the red arrow indicates flower primordium. (**d**) Fronds growing on medium supplemented with 20 µM SA showing lower amount of biomass after 15 days. (**e**) Magnification of the *red-squared* region in (**d**), showing flowers (red arrows); (**f**) Mature flower dissected from a budding pocket with pistil (red arrow), and two stamen (white arrows; anthers have shed).

Three commonly used duckweed media including liquid and/or semi-solid media H, E, and MH were used to study their influence on the rate of flowering. Two clones of *L. gibba*, 7741 and 5504, and one clone of *L. minor*, 7210 were incubated on these media under 16 h light period according to the previous reports for *L. gibba* G3^31, 38^. The fronds of all clones grew well on all media (Fig. 2a, controls). Immature flowers were found in more than 50% of the colonies of *L. gibba* 5504 and 7741, but no flower primordia could be observed in *L. minor* 7210 (data not shown). Mature flowers were hardly induced in any of the clones (Table 1, Fig. 2b,c), the highest rate of mature flowers obtained in solidified MH medium was approximately 2% for both of the *L. gibba* clones. First, we investigated photoperiod as a possible reason for the low percentage of mature flower formation. The clones were cultured under 8–16 h photoperiods. Immature flowers were induced at high frequencies in *L. gibba* 7741 and 5504 but not in *L. minor* 7210 under photoperiod ≥10 h (data not shown). However, most of these flower primordia in *L. gibba* were aborted and only a very low number of mature flowers were developed when grown in MH medium. Mature flowers were not observed at all in H and E media (Table 1). Flower primordia often co-existed with frond primordia in the same budding pocket (Fig. 2c).

**Table 1:** Influence of different basic media and salicylic acid on the rate of mature flower formation.

Clone	Nutrient medium	Time to first flower emergence (d)	Flowering rate (%)
*Lemna gibba* 7741	E	n/a	0
	H	n/a	0
	MH	20	1.7 ± 0.5
	E + 20 μM SA	10	51.0 ± 1.1
	MH + 20 μM SA	12	73.5 ± 3.5
*Lemna gibba* 5504	E	n/a	0
	H	n/a	0
	MH	20	2.3 ± 0.8
	E + 20 μM SA	14	6.5 ± 3.0
	MH + 20 μM SA	12	54.1 ± 4.2
*Lemna minor* 7210	E	n/a	0
	H	n/a	0
	MH	n/a	0
	E + 20 μM SA	n/a	0
	MH + 20 μM SA	n/a	0

Flower rate was evaluated 20 days after application of SA, the media were solidified with 0.6% agar. For the composition of the different Hoagland media E, H and MH cf. Table S1 (Supplementary information). Fronds were cultivated under 16 h light/8 h darkness. n/a = not applicable (no flowers occurred).

### SA promotes flower maturation

**Figure 3:**
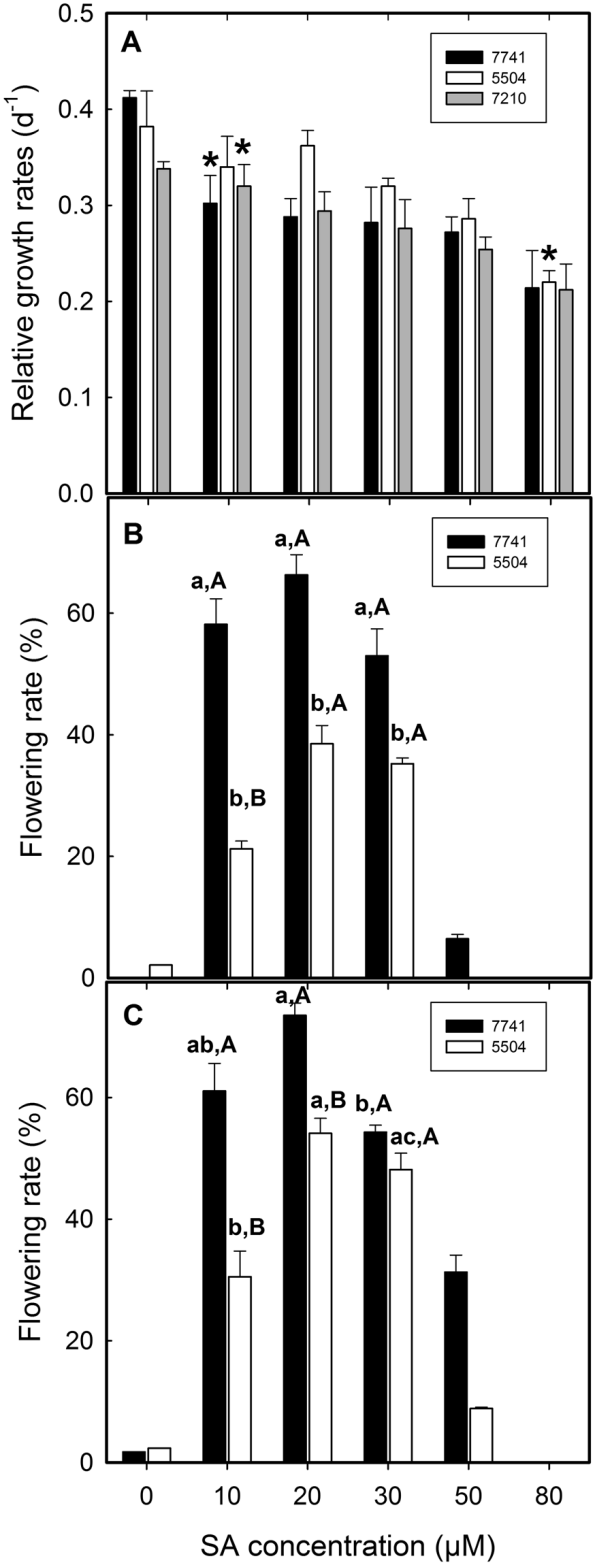
Dose-response effects of salicylic acid (SA) on growth and flowering rates. (**A**) Inhibitory effect of SA on the growth of *L. gibba* clones 7741 and 5504 and *L. minor* clone 7210 in semi-solid medium. Asterisks indicate the lowest concentration that significantly inhibited the growth of the three clones (students’s t-test, α < 0.05). (**B**) Effects of SA concentration in liquid medium on the flowering rates of *L. gibba* 7741 and 5504. (**C**) Effects of SA on solidified medium on the flowering rates of *L. gibba* 7741 and 5504. Note: Data are means of three independent experiments and are presented as mean ± standard error. Significance of differences in (**B**) and (**C**), were tested by one-way ANOVA (α < 0.05); Lower case letters mark significance of the effect of different SA concentrations, capical letters evaluate the differences in the response of both *L. gibba* clones. The results on *L. minor* 7210 in (**B**) and (**C**) were not presented because there was no stimulating effect.

When SA (e.g. 20 μM) was supplemented to E and MH media, the vegetative growth of all three clones, regardless of flower formation, slowed down although at different concentrations (cf. Fig. 2a,d; Fig. 3A). Mature flowers with one pistil and/or two stamens bending upward were observed under stereomicroscope (for *L. gibba* 7741 see Fig. 2e,f). Salicylic acid (SA) in MH medium resulted in higher flower induction rates than in E medium (Table 1). *Lemna gibba* 7741 had flowering rates of 51% and 74% in E + SA and MH + SA, respectively, while clone 5504 had flowering rates of 6.5% and 54%, respectively (Table 1). Even in the presence of SA, *L. minor* 7210 did not show any flower development.

**Figure 4:**
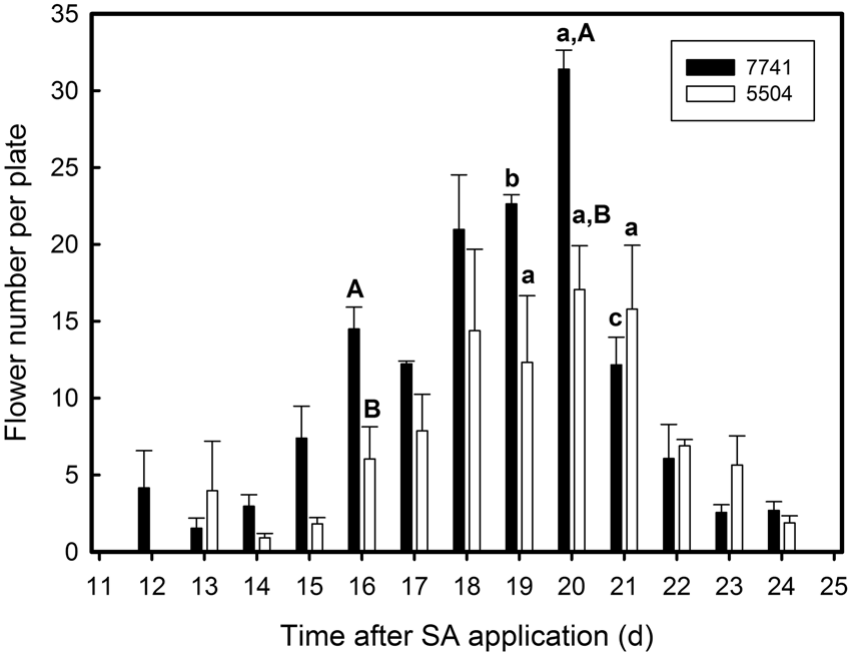
Temporal distribution of the number of flowering fronds. The fronds were cultured in 1/2 MH medium supplemented with 20 µM salicylic acid. Significance of differences from day 15 to day 21 after SA application were evaluated by ANOVA at a significance level of 0.05. Lower case letters indicate differences in the flower number over time, capital letters mark differences between *L. gibba* clones 7741 and 5504.

The first mature flowers were observed approximately 10−14 days after application of SA (Table 1, Fig. 4). The number of newly developed mature flowers scored daily, increased steadily in the following days until day 20, and declined thereafter in the two *L. gibba* clones. Approximately 70% of total mature flowers were obtained between day 17 and 21, with a peak around day 20 (Fig. 4). Approximately 17 and 31 flowers per plate on an average were obtained at day 20 in *L. gibba* 5504 and 7741, respectively. The flowering rates in the following experiments were presented always at day 20.

### Effect of SA concentration and agar solidification on flowering rate

Flowering rates were very low without SA in the nutrient medium (Table 1). When the lowest tested concentration of SA (10 μM) was supplemented to the liquid medium, the flowering rates already abruptly increased (Fig. 3B). The optimum SA concentration was 20 μM, although hardly any significant difference could be detected between 10 and 30 µM (in clone 5504 the rate was lower at 10 µM). At 20 µM SA flowering rates were 66% and 38% for *L. gibba* 7741 and 5504, respectively. When the SA concentration was increased further, flowering rates decreased (Fig. 3B). Almost no flowers were induced when the SA concentration was increased to 80 μM and frond death rate increased to approximately 70% (data not shown).

Semi-solid medium has not been tested previously for flower induction in duckweeds. The general effect of SA on the flowering rates in this medium was similar to that in the liquid medium, and the optimum SA concentration was also 20 μM (Fig. 3C). However, compared to the liquid media, the flowering rates in the semi-solid media were slightly higher in general and significantly higher at 20, 30 and 50 µM SA in clone 5504 (Fig. 3B,C). The highest flowering rate in the semi-solid media reached 74% and 54% for clones 7741 and 5504 at 20 µM SA, respectively.

### Difference in emergence of flowers and pollen characteristics in *Lemna gibba* 7741 and 5504

**Figure 5:**
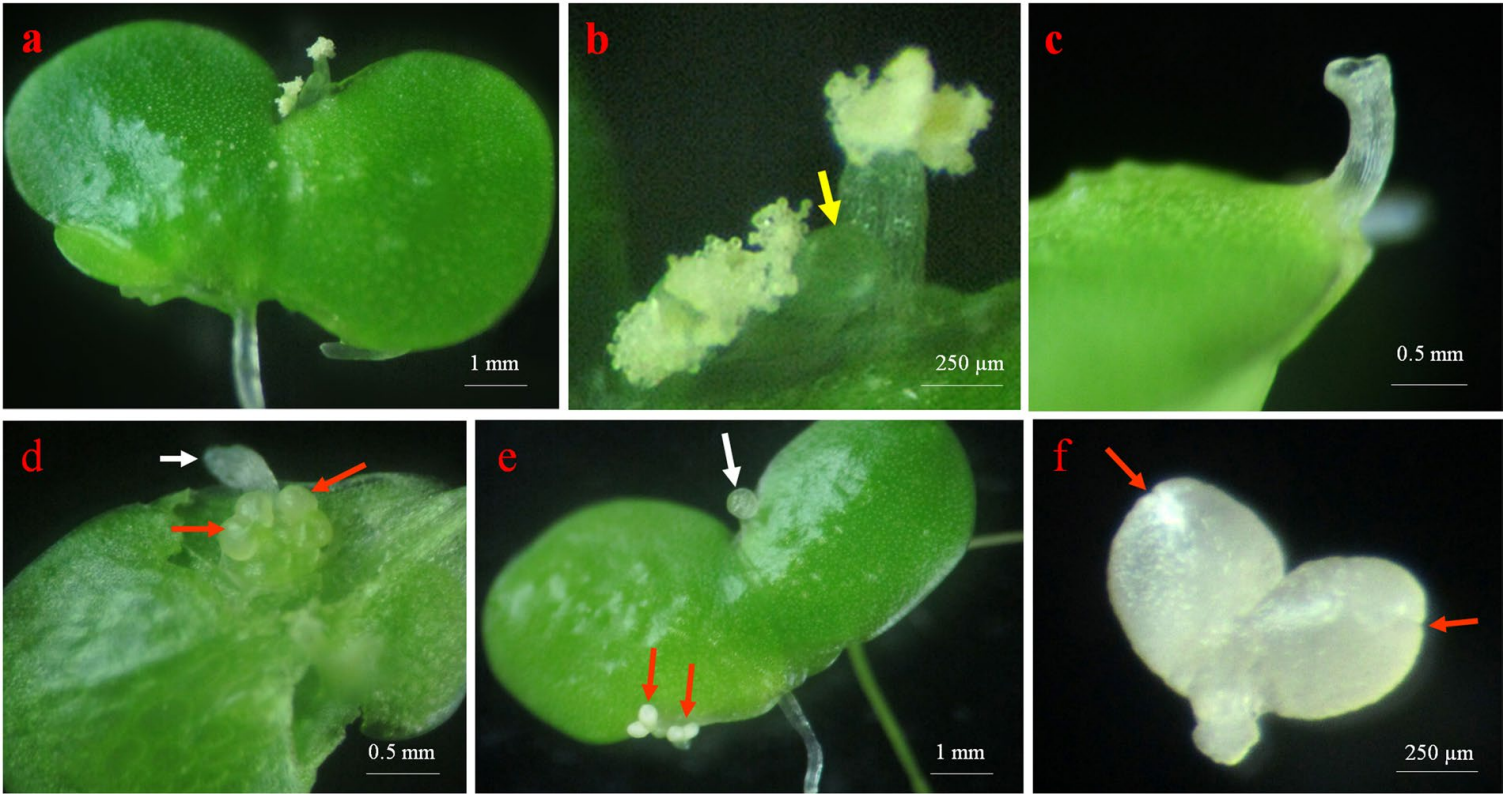
Flowering of *L. gibba* clones 7741 (**a**,**b**) and 5504 (**c**–**f**) under the influence of salicylic acid. (**a**) Flowering colony of clone 7741. (**b**) Magnification of the flower in (**a**), showing the stigma (yellow arrow) and pollens on the stamens. (**c**) Pistil emerged from the budding pocket of clone 5504 bending upward. (**d**) the same frond in (**c**) dissected from the ventral side to show the two stamens in the budding pocket (red arrows), the white arrow indicates the pistil. (**e**) Colony of 5504 with two stamens (red arrows) in one frond after the pistil withered, and a liquid droplet secreted by the pistil in another frond (white arrow). (**f**) Anther of clone 5504 that did not dihisce before withering (red arrows indicate the closed slits).

The stamens of *L. gibba* 7741 usually appeared ahead of the pistil (Fig. 5a), and the anthers released pollen grains rapidly after the appearance of pistil (Fig. 5b). Each anther of this clone contained approximately 210 pollen grains. In contrast, in *L. gibba* 5504 the pistil emerged first (Fig. 5c), and the stamens could be observed only by dissecting the budding pocket at this moment (Fig. 5d). A dew droplet was often observed on top of the pistil before stamens emerged (Fig. 5e), which was suggested to help in pollination. Moreover, the anthers of *L. gibba* 5504 were never observed to release pollen grains until they withered (Fig. 5f). Their pollen grains were released artificially with a dissecting needle, having a smaller number of approximately 180 grains per anther.

**Figure 6:**
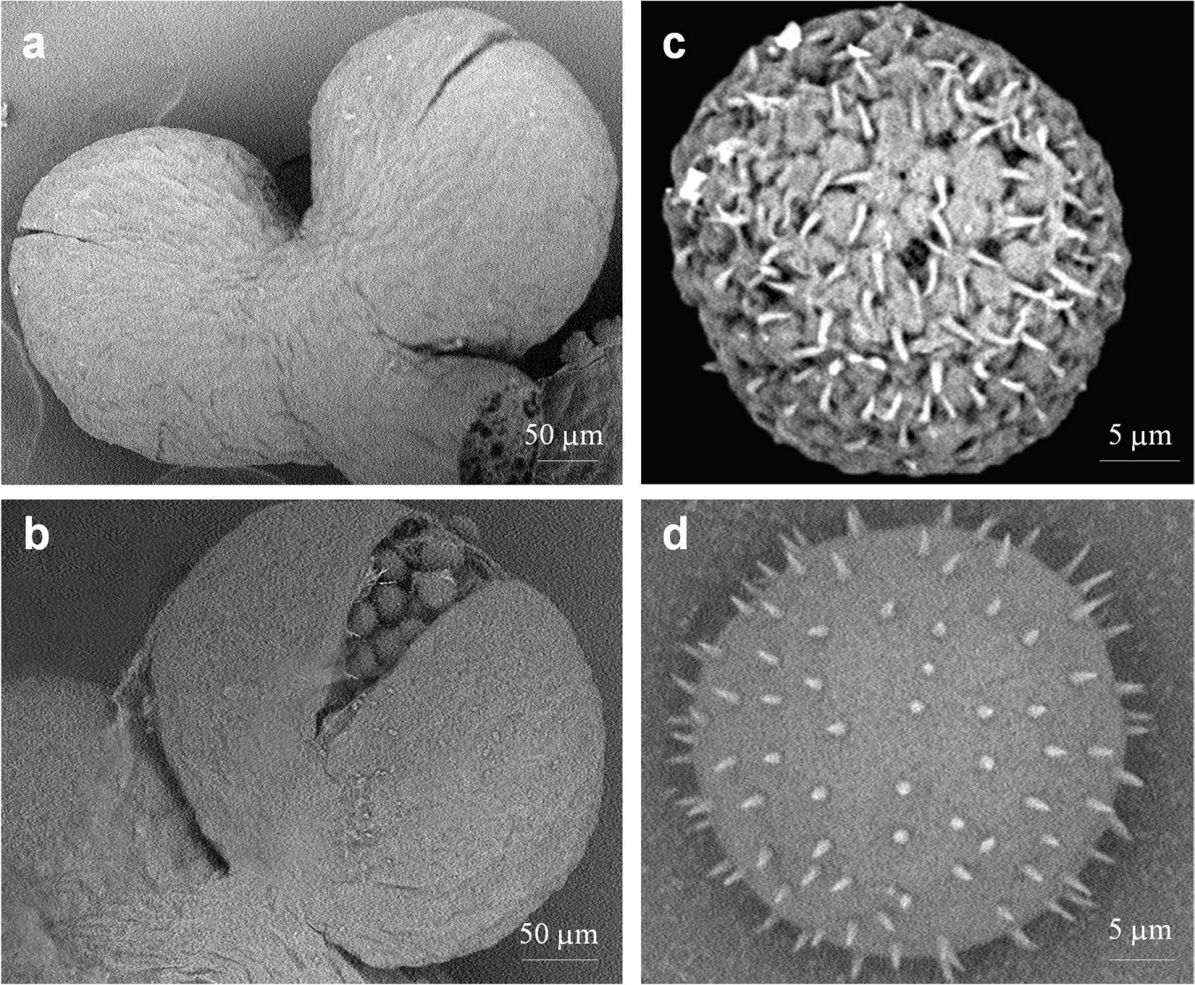
Scanning electron micrographs of anthers and pollen grains. (**a**) Anther of *L. gibba* 5504 showing the closed slit. (**b**) Anther of *L. gibba* 7741 with opened slit. (**c**) Pollen grain of clone 5504 showing pineapple-like exine and tilted spines. (**d**) Pollen grain of clone 7741 showing the smooth exine and uniformly distributed spines.

Scanning electron microscopy indicated that the slits of the mature anther of *L. gibba* 5504 remained closed (Fig. 6a), while that in the anthers of *L. gibba* 7741 was widely open (Fig. 6b). The artificially released pollen grains of *L. gibba* 5504 had pineapple-like exine with tilted spines of 0.6 to 1.3 μm in length (Fig. 6c). The sizes of the pollen grains varied in diameter from 15 to 21 μm. In contrast, the pollen grains of *L. gibba* 7741 had smooth exine with uniformly and vertically distributed spines (Fig. 6d). The sizes of 7741 pollen grains were relatively uniform with a diameter between 20 to 22 μm.

### Pollen viability test by chemical staining and germination

**Figure 7:**
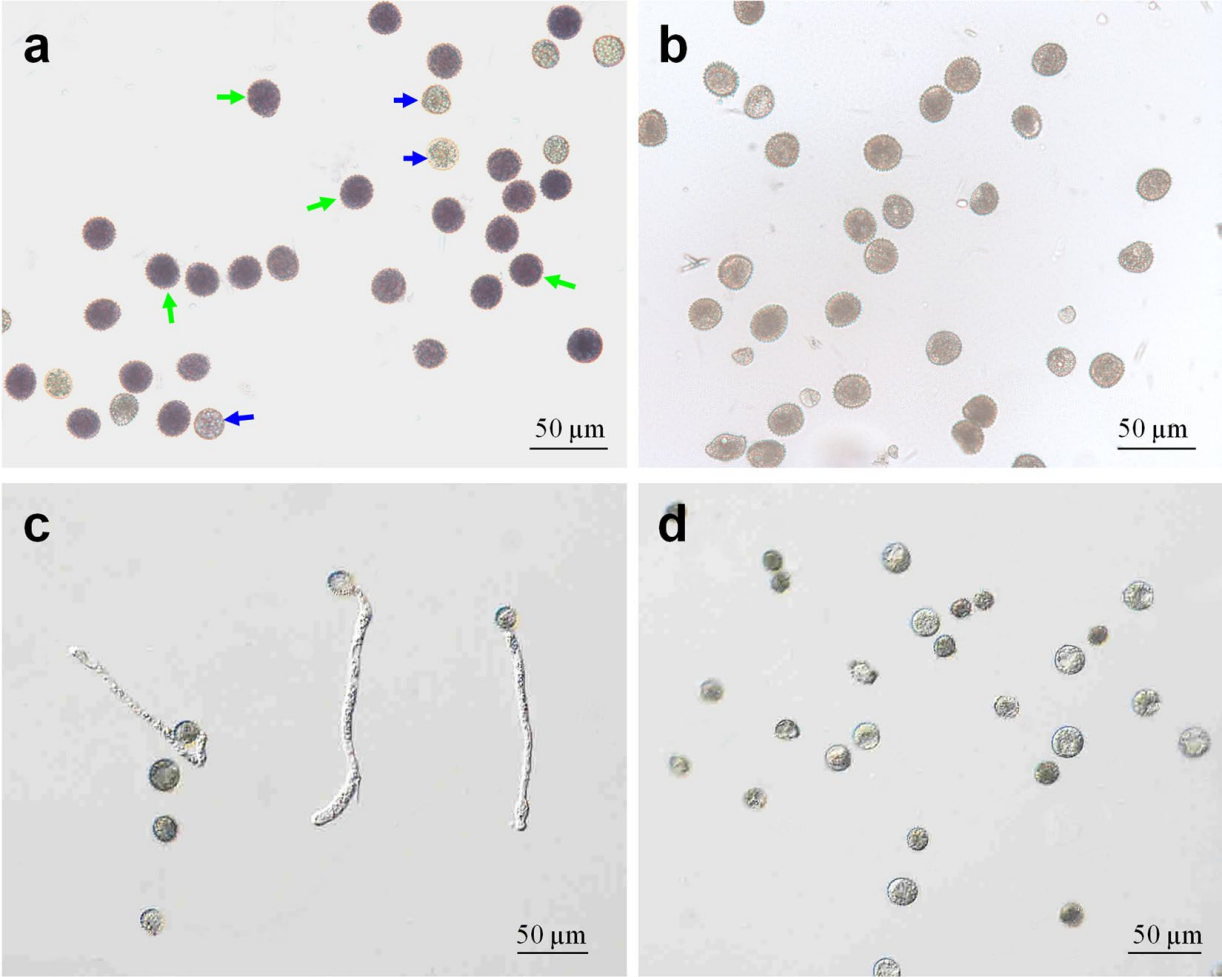
Viability test of pollen grains from *L. gibba* 7741 and 5504. (**a**) Pollen grains from clone 7741 stained with MTT solution. (**b**) Pollen grains from clone 5504 stained with MTT solution. (**c**) Germinating pollen grains of clone 7741. (**d**) Lack of germination of pollen grains of clone 5504 after 16 h of incubation under germination conditions. The green and the blue arrows indicate the viable and the non-viable pollen grains, respectively.

Several chemical staining methods were tested to distinguish viable and non-viable pollen grains in duckweeds. The MTT method was most effective, staining viable pollen grains of *L. gibba* 7741 violet-purple whereas dead pollen grains (killed by heating) lacking almost any stain (Supplementary Fig. S2, Supporting Information). Pollen grains of *L. gibba* 5504 and 7741 were then stained in parallel experiments (Fig. 7a,b). Most of the pollens of clone 7741 were stained dark violet (approximately 67 ± 3%; Fig. 7a), and were considered viable. Approximately 20% of the pollen grains of clone 7741 were not stained and were considered non-viable. The rest of the pollen grains (approximately 10%) were stained pale violet, and their viability was therefore uncertain. In contrast, none of the pollen grains of clone 5504 were stained dark violet (Fig. 7b).

The viability of pollens was further tested by germination experiments. Some of the pollen grains of clone 7741 germinated within five minutes, and germination frequency was approximately 40% as counted 3 h later (Fig. 7c), whereas none of the pollen grains of clone 5504 germinated even if the incubation time was prolonged to 24 h (Fig. 7d). Therefore, we concluded from both experiments (Fig. 7b,d) that pollen of clone 5504 was sterile.

### Seed formation following pollination

**Figure 8:**
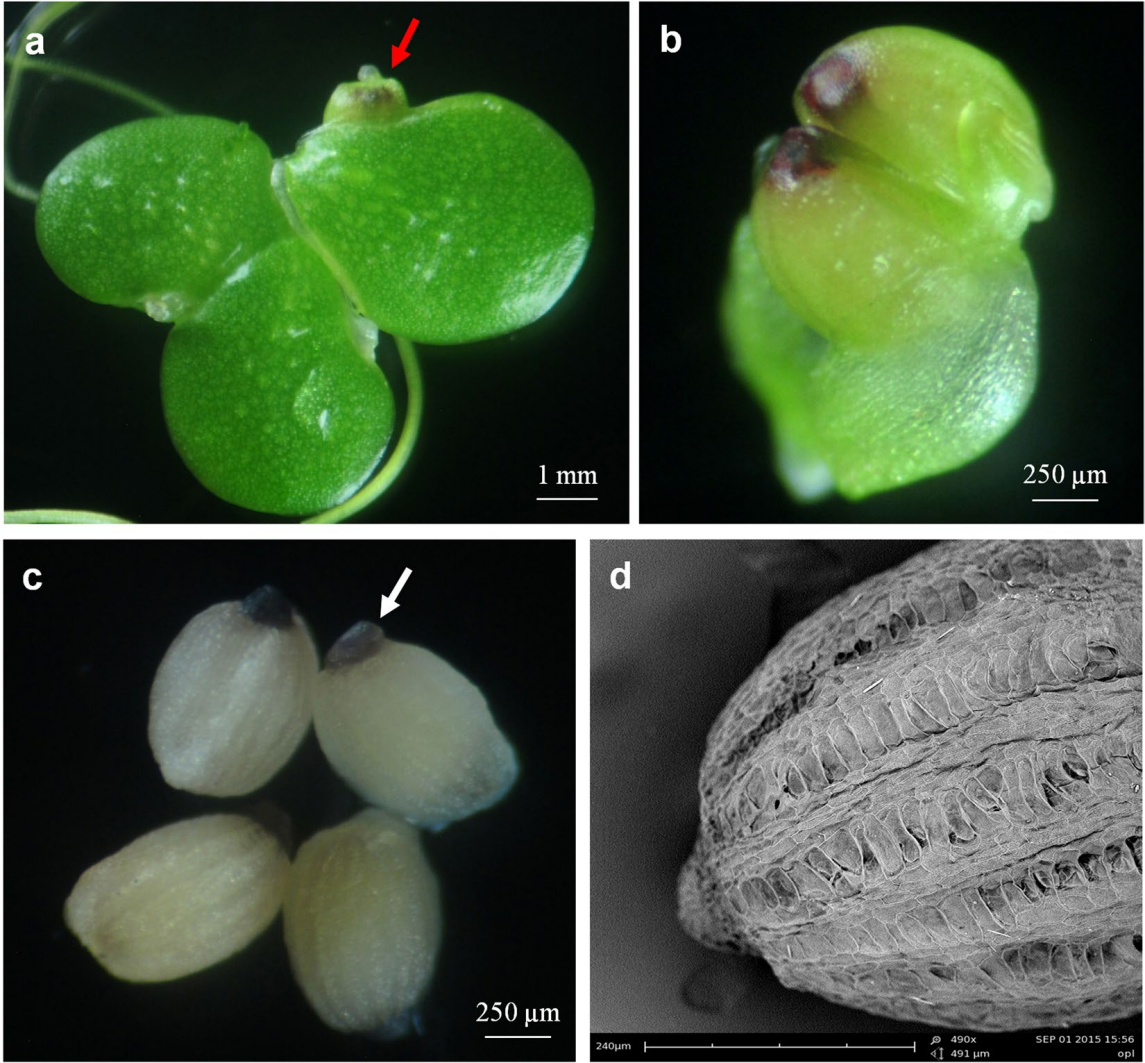
Seeds of *L. gibba* 7741. (**a**) Colony with an immature fruit (red arrow). (**b**) Immature fruit with two seeds. (**c**) Mature seeds with purple operculum (white arrow). (**d**) Scanning electron graph of a seed showing the longitudinal-ribs.

Natural pollination of flowers of *L. gibba* in the culture plates rarely produced seeds. Artificial pollination between individuals of *L. gibba* 7741, however, produced seeds (Fig. 8a) with a setting rate of approximately 20–30%. Two or three seeds were most often observed enclosed in the thin-layer pericarp of the fruit (Fig. 8b), one or four seeds were observed occasionally in one fruit. The mature seeds were white and oval with a purple operculum to the posterior end (Fig. 8c). Scanning electron microscopy showed that the seed coat was covered with longitudinal-ribs (Fig. 8d).

In contrast to clone 7741, no seeds were obtained from clone 5504 after natural and artificial pollination between individuals of this clone.

### Artificial cross-pollination between *L. gibba* clones and validation of hybrids

**Figure 9:**
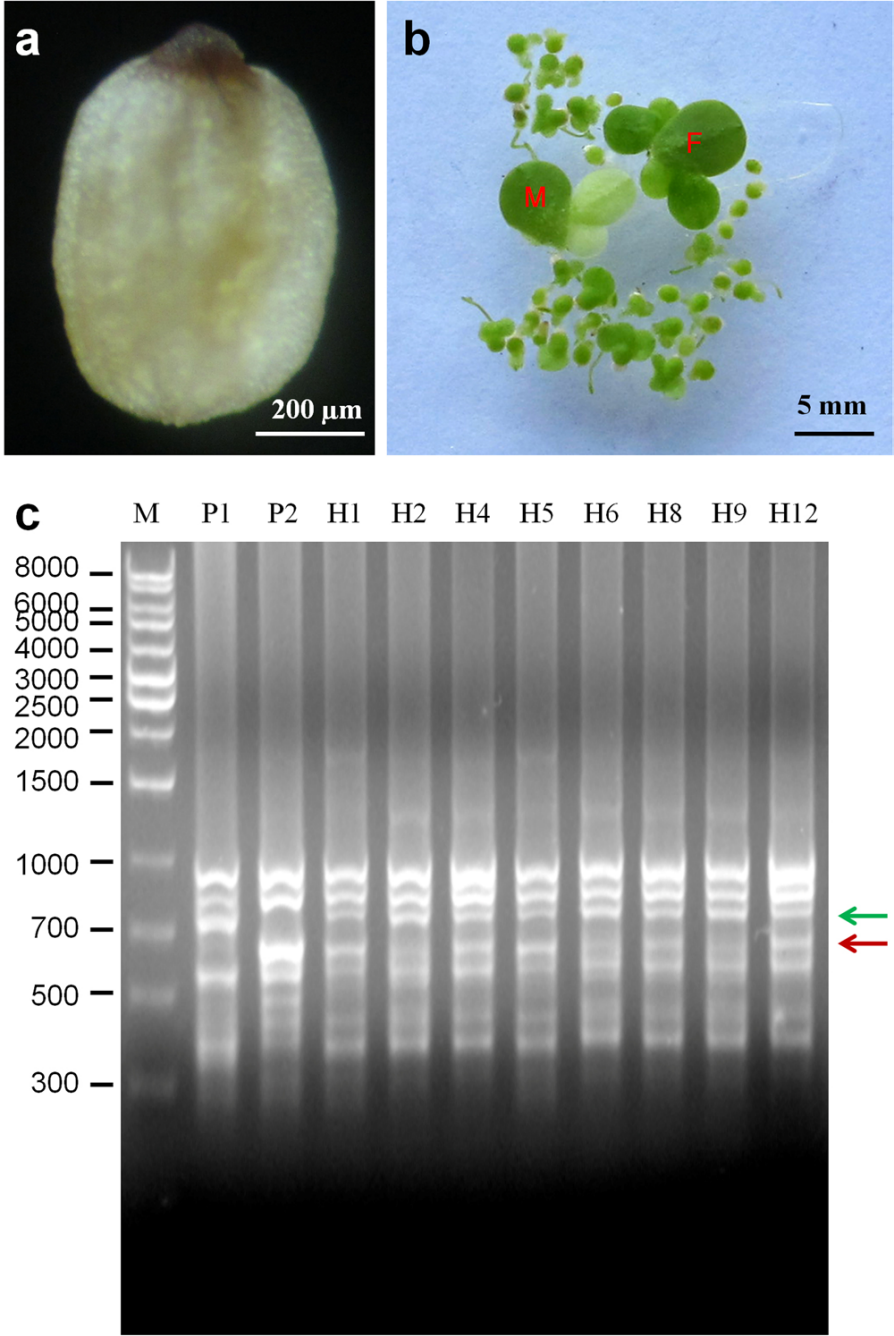
Germination of hybrid seeds and ISSR validation. (**a**) A typical hybrid seed from crossing between clones 5504 and 7741. (**b**) Hybrid clones five days after germination, their female (F) and male (M) parents are used as size controls. (**c**) ISSR validation of the hybrid clones; M, molecular weight marker (Axygen 1 kb ladder); P1, male parent 7741; P2, female parent 5504; H1 through to H12, hybrid clones; the green arrow indicates 7741-specific band, the red arrow indicates 5504-specific band.

Artificial cross-pollination of the stigmas of clone 7741 using pollen grains of clone 5504 did not result in any seed formation. However, artificial pollination of the stigmas of clone 5504 using the pollen grains of clone 7741 produced intraspecific hybrid seeds. These seeds were morphologically identical to those resulted from self-pollination of clone 7741 (Fig. 9a). Also, the hybrid seeds germinated normally at the same rate as those formed after self-fertilization: seeds from clone 7741 alone germinated to 100% (100 seeds tested), and hybrid seeds germinated to 97% (35 seeds tested). Hybrid colonies were formed after germination as shown in Fig. 9. The initial hybrid fronds were small compared to their parents (Fig. 9b); however, the subsequent fronds grew larger gradually until they reached the size of their parents.

In order to prove that indeed hybrids were formed between the paternal clone 7741 and the maternal clone 5504, the parental and hybrid clones were investigated by ISSR by testing 50 primers. Primer B825 showed parent-specific bands and was used to test the assumed hybrid clones (Fig. 9c). Results indicated that both parental bands could be clearly detected in the hybrid lines, therefore, the hybrid lines inherited the genetic properties of both parents (Fig. 9c).

## Discussion

### Flower induction in *Lemna gibba* but not in *Lemna minor*

In order to develop a method for efficient flower induction, which serves the purpose of cross-pollination, we measured the flowering rate as the number of colonies with mature flowers divided by the number of total colonies. The previously used number of immature flowers^30, 38, 39^ is not useful for subsequent pollination and seed formation experiments. We successfully induced mature flowers at high frequencies for *L. gibba* clones 7741 and 5504, respectively. No flower induction was observed, under any of the conditions tested, in *L. minor* 7210, which suggested that different duckweed species responded differently to SA induction and may need different methods to induce flowering, e.g., in the case of *L. minor*, ethylenediamine-N,N’-bis (2-hydroxyphenylacetic acid) (EDDHA) is a successful flower-inducer^40^. Although no flowering was induced in *L. minor* 7210 by SA, its growth rate was reduced significantly, which indicated that growth inhibition was not a result of flowering. Flowering and vegetative growth are not always competitive in duckweeds, for example, *Wolffia microscopica* was reported to grow very rapidly during continuous flowering season^41^.

In the previous reports, authors had considered immature flowers to quantify flower induction^30, 38, 39^. However, this parameter is not useful for subsequent pollination and seed formation experiments as most of the immature flowers were aborted during subsequent development. In the present paper a method was developed to induce the formation of mature flowers at high rates, calculated as the number of colonies with mature flowers divided by the number of total colonies. Without SA in the MH or E media, flower primordia (i.e. immature flowers) were induced in *L. gibba* 7741 and 5504 at high frequencies. However, mature flowers were obtained only at very low percentages (ca. 2% in MH medium). Supplementation of media with SA increased the rates of formation of mature flowers dramatically, for both *L. gibba* 7741 and 5504. The results in semi-solid MH medium with SA were even slightly higher than in liquid medium. Formation of high percentage of mature flowers is a prerequisite for the production of seeds.

It was reported that ammonium in the Hutner’s medium inhibited the flower primordia formation in *L. gibba* G3^30, 38^. This inhibition effect could be reversed by addition of SA and hexacyanoferrate (III) to the medium^30^. However, we obtained high percentage of flower primordia in *L. gibba* 7741 and 5504 even in the ammonium containing MH medium, which was more than 50%, respectively. Therefore, ammonium may not be an inhibition factor for flower primordial differentiation at least in clones 7741 and 5504.

Moreover, the flowering rates presented here were based on the mature flower counts at the end of the experiment (20 days), when flower percentage was at the climax (Fig. 3), ignoring the flowers matured before the end of the experiments. Therefore, the actual flowering rates were even higher than what are presented here. We concluded that the induction of mature flowers with SA is a useful technique for later cross-pollination and breeding.

### Evaluation of pollen viability

In order to produce seeds, pollen must be viable. Chemical staining is a widely used method to examine pollen viability^42–44^. We used several staining reagents to test the viability of *L. gibba* pollen grains and found that the MTT staining was the most effective (Supplementary Fig. S2). Heat-killed pollens were recommended previously as a negative control^44^. We adopted this method to prepare dead pollens. However, the spines or echinates were removed from the exine by the heat-killing process (Supplimentary material, Fig. S2), which resulted in varied morphology, and therefore need to be improved in the future. Regardless of this, pollens of *L. gibba* 7741 clearly showed viability as tested by staining and pollen germination. This was not the case for pollens of *L. gibba* 5504 (Fig. 7). Thus, vital pollens were produced from mature flowers in clone 7741 but not in clone 5504.

### Hybrid vigor and male sterility

It has been shown in the present paper that the success of spontaneous self-fertilization can be greatly improved by artificial self-fertilization and that even intraspecific cross-fertilization between different clones of *L. gibba* is possible to succeed with high rate. Artificial pollen transfer from clone 7741 to stigmas of clone 5504 resulted in fertilizaiton and formation of seeds. These seeds germinated normally and formed new vegetative plants. To prove that these plants were intraspecific hybrids formed by clone 7741 as pollen-donor and 5504 as pollen acceptor, parent plants and seedlings were analyzed by ISSR. The presence of parent-specific bands in the seedlings demonstrated that these plants are indeed hybrids. Artificial transfer of pollens from clone 5504 to stigmas of clone 7741 did not result in fertilization. We conclude that *L. gibba* 5504 is male-sterile. To the best of our knowledge, this is the first report about male-sterility in the duckweed family. Our conclusion is based on the following results: First, the emergence of the stamens of clone 5504 lagged behind the pistil. Second, the anthers of clone 5504 were not ruptured and pollen grains were not released. Third, the pollen grains of clone 5504 had an underdeveloped and pineapple-like exine with tilted spines. Fourth, they were not stained by MTT, and finally, these pollens did not germinate, and did not result in seeds by pollinating on stigmas of both clones 5504 and 7741.

Male sterility is one of the bases in commercialization of hybrid vigour and has been widely used in hybrid seed production of maize^45, 46^, rice^47, 48^, wheat^49^, cotton^50^, oilseed rape^51^, and many other crops^52^. The discovery of a male-sterile line of duckweed may be useful in future application of hybrid vigour in different applications of duckweed.

## Methods

### Plant materials and culture media

Three axenic clones, *Lemna gibba* L. 5504, 7741 and *L. minor* 7210, were used. Clone 5504 was isolated from the Innovation River in Pudong, Shanghai, China, with an original accession number SH0204^53^ and maintained at the Biological Collection Centre of the Institute of Tropical Bioscience and Biotechnology, Chinese Academy of Tropical Agricultural Sciences, as well as at the Rutgers Duckweed Stock Cooperation at Rutgers University, New Jersey, USA under international accession number 5504. Clone 7741 was a standard *L. gibba* clone derived from clone G3^35^, the later was originally isolated from Sicilia, Siracusa, Italy by R. Kandeler, Vienna, Austria. Clone 7210 was identified as *L. minor* (Supporting Information, Fig. S1). It was originally isolated in Japan, maintained in Landolt’s collection, Zurich, Switzerland and obtained from the Rutgers Duckweed Stock Cooperative. The identities of these clones were verified by phylogenetic analysis based on their *atpF-atpH* intergenic sequences and their *rps16* intron sequences as suggested by Borisjuk *et al*.^36^ (Supporting Information, Fig. S1).

The components in the three basic nutrient media used in this study are provided in Table S1 (Supporting Information). All nutrient media were supplemented with 29 mM sucrose with or without 6 g L^−1^ agar (Biotechnology grade, Beijing Solarbiao Science & Technology co., Ltd, Beijing, China) for solidification. Media were autoclaved and the SA stock solution was filter sterilized (0.22 µm filter units; Millipore, USA).

### Observation of flower primordium and determination of critical day length

To observe flower induction and to determine critical day length, fronds were cultured in MH medium under 8 h light/16 h darkness for 20 days, then transferred to fresh MH or E-medium and continued to culture under 8, 9, 10, 12, 14, and 16 h photoperiod for 20–30 days. The budding pockets were dissected and observed under a dissecting microscope.

### Effects of SA on flower induction

Three-frond colonies of the above mentioned clones were cultured in three replicates on semi-solid MH medium supplemented with 29 mM sucrose and 20 μM SA, and cultivated under 16 h photoperiod at 40 μmol · m^−2^ · s^−1^ photosynthetic active radiation (PAR) at 25 ± 1 °C. Each replicate contained 10 plates, and each plate contained five colonies. In contrast to previous reports, only fronds with mature flowers were considered as flowering, which is in accordance with the purpose of the investigation. Mature flowers were counted every day from their first emergence until day 24.

To study the effect of the SA concentration on the flowering rate, three-frond colonies were inoculated with threefold replicates in the MH medium supplemented with different concentrations of SA (0, 10, 20, 30, 50, and 80 μM) and cultivated under 16 h daily photoperiod at 40 μmol · m^−2^ · s^−1^ PAR at 25 ± 1 °C. Flowering rates were calculated 20 days after inoculation. The flowering rate was defined as the total number of plants (frond colonies) with mature flowers at the time of counting divided by the total number of plants inspected.

### Effects of SA on frond growth rate

Three-frond colonies were inoculated in three replicates on the basal medium supplemented with different concentrations of SA (0, 10, 20, 30, 50, and 80 μM) and incubated under 16 h day photoperiod at 40 μmol · m^−2^ · s^−1^ PAR at 25 ± 1 °C. Frond number of each sample was counted seven days after inoculation. The relative growth rate (RGR) was calculated as described previously^54^: RGR = (lnN_2_ − lnN_1_)/t, where N_1_ represents the initial frond number, N_2_ represents the final frond number, and t represents the days of incubation.

### Estimation of pollen grains in the anthers

To estimate the quantity of pollen grains in the anthers, 30 mature anthers from randomly chosen colonies were put in three 1.5 mL centrifuge tubes with 10 anthers in each tube and broken with a vaccination needle. The broken anthers were then treated with 0.5 mL 2.5% pectinase (Y23, Yakult Pharmaceutical Industry Co., Ltd., Tokyo, Japan) solution for 4 h at 28 °C, followed by dilution with 0.5 mL 2.5% sucrose solution. The centrifuge tubes were then vortexed for 10 sec. The resulting pollen suspension was spread on glass slides in 5 μL droplets, and the pollen grains in each droplet were counted under a light microscope (Axioskop 40, Zeiss, Jena, Germany) with triplicates for each sample. Pollen grain quantity in each anther was calculated as the average number of pollen grains in each 5 μL droplet × the total volume of pollen suspension in one tube (1000 μL)/the volume of each droplet (5 μL)/the number of anthers in each tube (10).

### Pollen viability tests by chemical staining

Four different staining methods were tested for their effectiveness to distinguish between viable and non-viable pollen (Trognitz 1991; Khatun & Flowers 1995). As control, pollens incubated at 80 °C for 2 h were considered dead^55^. The staining reagents included: (1) 2,3,5-triphenyl tetrazolium chloride (TTC), (2) 2,5-diphenyl monotetrazolium bromide (MTT), (3) 5-bromo-4-chloro-3-indoyle-β-galactoside (X-gal) (Rodriguez-Riano & Dafni 2000), and (4) fluorescein diacetate (FDA) (Kearns & Inouye 1993).

The MTT reagent was finally selected. Mature anthers were placed on a glass slide and incubated with 5 μL MTT solution. The working solutions of MTT contained 1% staining reagent with 5% sucrose that was added to the anthers. Pollen grains were released into the solution by breaking the anthers with a vaccination needle. After incubating for 10 min, the pollen grains were observed under a light microscope (Axioskop 40, Zeiss, Jena, Germany), and the viability was evaluated by the colour of the pollen. Ten anthers were examined per clone. The pollen viability is given as percentage of the number of viable pollen grains to the total number of pollen grains observed.

### Pollen viability test by germination

Germination medium (MH medium supplemented with 1% sucrose) was spread on glass slides to a thin layer. Pollen grains were then inoculated by scratching the anthers on the surface of the medium. The germination of the pollen grains was observed under microscope at intervals of five minutes.

### Scanning electron microscopy

Mature anthers were fixed in formaldehyde-acetic acid-ethanol solution (FAA, 100 ml solution contains 5 ml 40% formaldehyde, 5 ml glacial acetic acid, and 90 ml 70% ethanol) at 4 °C overnight, and then dehydrated in a series of tert-butyl alcohol (30%, 50%, 70%, 80%, and 90%) for 15 min at each concentration. The anthers were dehydrated with 100% tert-butyl alcohol three times for 1 hour. The dehydrated anthers were then freeze-dried and inspected with a scanning electron microscope (FEI Phenom, Phenom Wold, Eindhoven, Netherlands). Pollen grains were released from the dehydrated anthers with a vaccination needle and suspended in tert-butyl alcohol. Aliquots of the pollen suspension were then spread over glass slides, adhered to electron microscopy sample tables (FEI Phenom), and observed with a scanning electron microscope (FEI Phenom). Twenty pollen grains from each clone were randomly chosen to measure their size and to study their morphology.

### Artificial cross-pollination under stereomicroscope

Flowering fronds of *L. gibba* 7741 and 5504 were transferred to fresh semi-solid MH medium supplemented with 29 mM sucrose and were incubated at 40 μmol . m^−2^ . s^−1^ PAR and 25 ± 1 °C for 24 h. Artificial pollination was then performed in the tissue culture hood under a stereomicroscope. Pollen grains of clone 7741 were pollinated on the stigma of clone 5504 under a stereomicroscope by moving the whole colony with tweezers towards the stigma. The pollinated fronds were then transferred to fresh plates containing MH medium supplemented with 29 mM sucrose and 20 μM SA and incubated under 16 h daily photoperiod of 40 μmol · m^−2^ · s^−1^ PAR at 25 ± 1 °C. Seeds were harvested after 20 days. Similar experiments were carried out by transferring pollens of clone 7741 on to the stigma of clone 5504 in order to test the possibility for intraspecific cross-pollination between different clones.

### Seed germination and validation of hybrid formation

Fresh seeds were punctured on the seed coat with a scalpel, and were incubated in MH medium under 16 h daily photoperiod of 40 μmol · m^−2^ · s^−1^ PAR at 25 ± 1 °C. Each line that was vegetatively propagated from one seed was considered as a new clone.

DNA was isolated using a genomic DNA extraction kit (Tiangen Biotech Co. LTD, Beijing, China) according to the reccommended protocol. Fifty primers that were selected from the universal inter-simple sequence repeat (ISSR) primers^56^ were synthesized and used to distinguish genetic differences between parental clones 7741 and 5504. One primer (B825, 5′ ACACACACACACACACT 3′) showed clone-specific bands and was used to validate the genetic background of the hybrid clones. The PCR reactions were performed at 94 °C for 4 min, followed by 30 cycles of 94 °C for 30 sec, 48 °C for 30 sec, and 72 °C for 1 min. The PCR products were separated on 1.5% agarose gel, stained with Goldview Nucleic Acid Gel Stain (Solarbio, Beijing, China), and visualized using the gel imager Tanon 410 (Tanon, Shanghai, China).

## Electronic supplementary material

Supplementary Information
