# Supplementary material for: Flower induction, microscope-aided cross-pollination, and seed production in the duckweed Lemna gibba with discovery of a male-sterile clone
Source: Sci Rep. 2017 Jun 8;7:3047. doi: 10.1038/s41598-017-03240-8 (PMC5465175; doi:10.1038/s41598-017-03240-8)
Supplement: Supplementary file 1 — Supplementary Information [file 41598_2017_3240_MOESM1_ESM.pdf]

Supplementary Information

Flower induction, microscope-aided cross-pollination, and seed production in the duckweed *Lemna gibba* with discovery of a male-sterile clone

Lili Fu[1,*], Meng Huang[1,*], Bingying Han[1], Xuepiao Sun[1], K. Sowjanya Sree[2], Klaus-J Appenroth[3], Jiaming Zhang[1]

[1]*Institute of Tropical Bioscience and Biotechnology, MOA Key Laboratory of Tropical Crops Biology and Genetic Resources; Hainan Bioenergy Center, CATAS, Haikou, Hainan Province, 571101, China;*

[2]*Department of Environmental Science, Central University of Kerala, RSTC, Padanakkad-671314, Kerala, India;*

[3]*Institute of Plant Physiology, University of Jena, Dornburger Str. 159, 07743 Jena, Germany*

*These authors contributed equally to this work.

Correspondence and requests for materials should be addressed to J.Z. (zhangjiaming@itbb.org.cn)

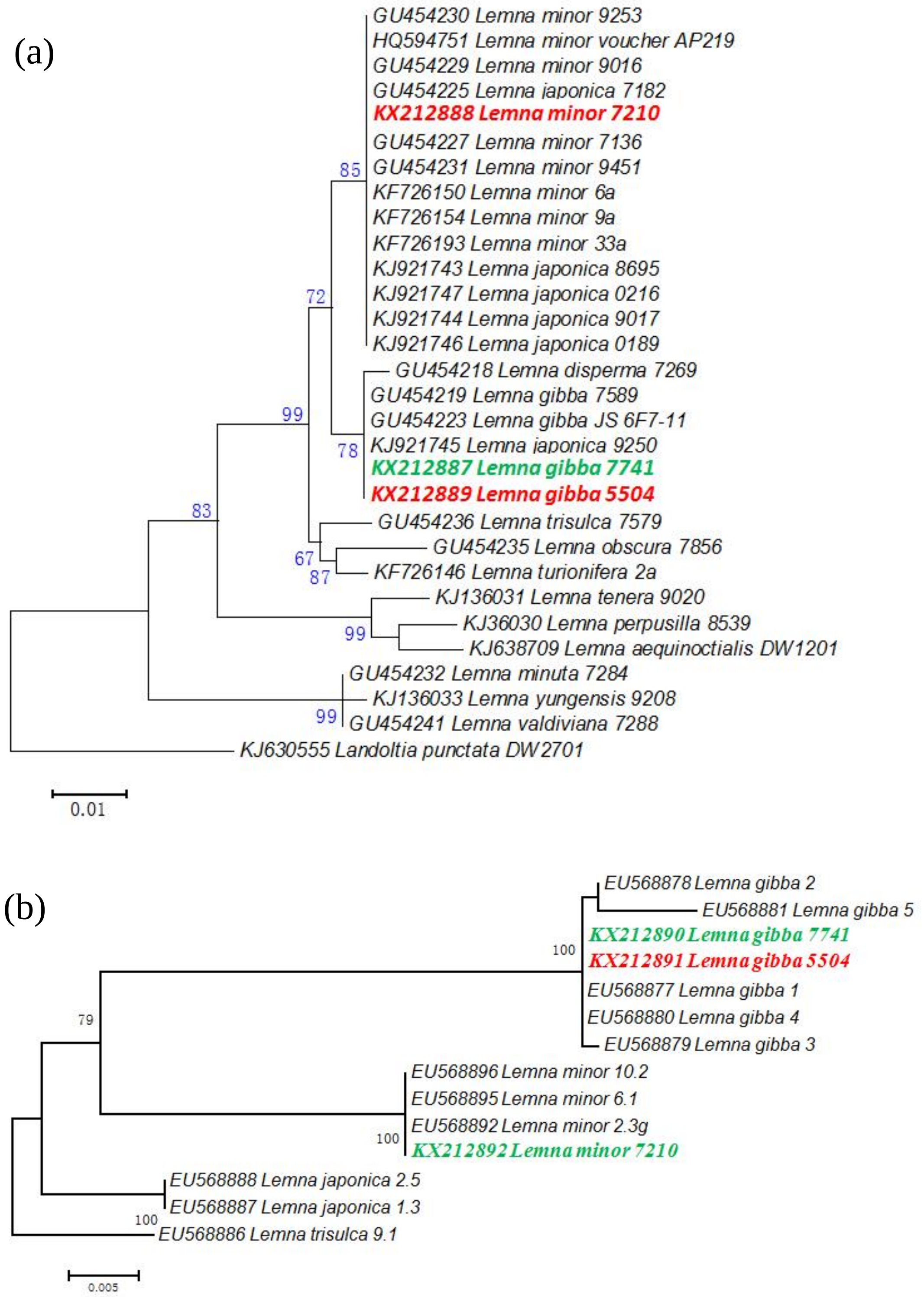

Figure S1. **Phylogenies inferred with the chloroplast *atpF-atpH* intergenic spacer (a) and *rps16* intron (b) sequences in *Lemna* species**. Evolutionary analyses were conducted in MEGA7[1]. The evolutionary history was inferred by using the Maximum Likelihood method based on the Tamura-Nei model. The trees with the highest log likelihood are shown. The bootstrap values are shown next to the branches. The trees are drawn to scale and rooted with *Landoltia punctata* DW2701 (**a**) or *Lemna trisulca* 9.1 (**b**). The scale bars represent 0.01 (**a**) or 0.005 (**b**) substitutions per site.

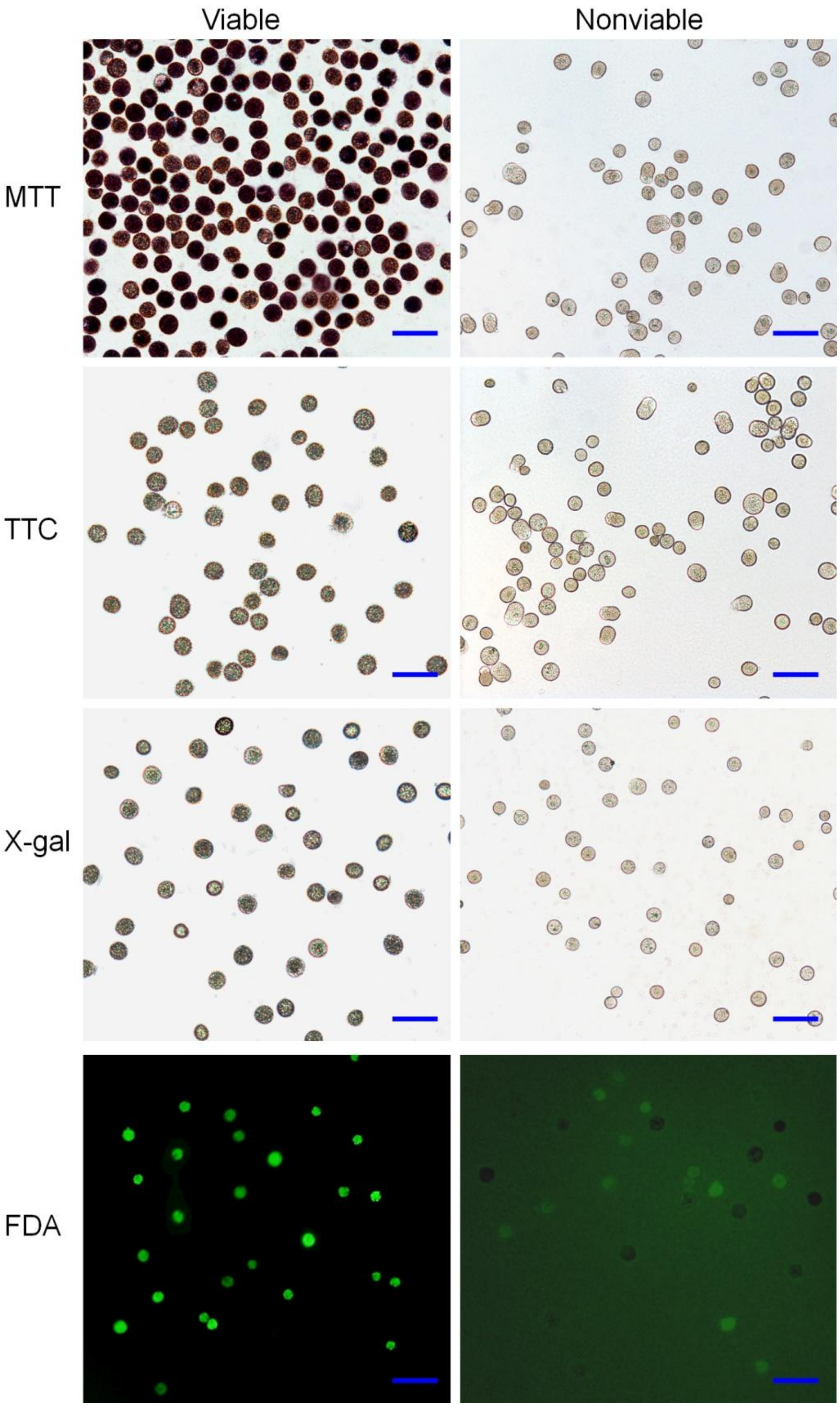

Figure S2. **Different chemical staining of fresh (left side) and dead (heat inactivated; right side) pollen grains from clone 7741**. The staining methods were listed to the left of each row. Scale bars represent 50 μm.

Table S1. Basal salts in the media used in this study

Chemicals	H (mmol/L)	MH (mmol /L)	E (mmol /L)
NH_4NO_3		80	
KNO_3	5	5	15
KH_2PO_4	1	1	5
$MgSO_4.7H_2O$	2	2	2
$Ca(NO_3)_2.4H_2O$	5	4	5
H_3BO_3	0.046	0.1	0.046
$MnCl_2.4H_2O$	0.009		0.018
$MnSO_4.H_2O$		0.1	
$ZnSO_4.7H_2O$	0.0008	0.03	0.0008
$CuSO_4.5H_2O$	0.0003	0.0001	0.0003
$H_2MoSO_4.H_2O$	0.0001		
$Na_2MoSO_4.2H_2O$		0.001	0.0005
KI		0.005	
$CoCl_2.6H_2O$		0.0001	
$FeSO_4.7H_2O$	0.1	0.1	
$FeCl_3.6H_2O$			0.02
Na_2EDTA	0.1	0.1	
EDTA			0.03
tartaric acid			0.02
pH	5.7	5.7	4.6

Note. H—Hoagland medium [2]
MH—Modified Hoagland medium
E—E-medium [3]

References:

1 Kumar, S., Stecher, G. & Tamura, K. MEGA7: Molecular Evolutionary Genetics Analysis version 7.0 for bigger datasets. *Molecular Biology and Evolution* **33**, 1870-1874 (2016).

2 Hoagland, D. R. & Arnon, D. I. The water-culture method for growing plants without soil. *California Agricultural Experiment Station Circular* **347**, 1-32 (1950).

3 Cleland, C. F. & Briggs, W. R. Flowering responses of the long-day plant *Lemna gibba* G3. *Plant Physiology* **42**, 1553-1561 (1967).
